# The reciprocal relationship between compounding awareness and vocabulary knowledge in Chinese: a latent growth model study

**DOI:** 10.3389/fpsyg.2015.00440

**Published:** 2015-04-15

**Authors:** Yahua Cheng, Liping Li, Xinchun Wu

**Affiliations:** School of Psychology, Beijing Normal UniversityBeijing, China

**Keywords:** compounding awareness, vocabulary knowledge, latent growth modeling, reciprocal relationship, Chinese children

## Abstract

The aim of this study is to examine the developmental relationship between compounding awareness and vocabulary knowledge from grades 1 to 2 in Chinese children. In this study, 149 Chinese children were tested on compounding awareness and vocabulary knowledge from Time 1 to Time 4, with non-verbal IQ, working memory, phonological awareness, orthographical awareness, and rapid automatized naming at Time 1 as control variables. Latent growth modeling was conducted to analyze the data. Univariate models separately calculated children's initial levels and growth rates in compounding awareness and vocabulary knowledge. Bivariate model was used to examine the direction of the developmental relationships between the two variables with other cognitive and linguistic variables and the autoregression controlled. The results demonstrated that the initial level of compounding awareness predicted the growth rate of vocabulary knowledge, and the reverse relation was also found, after controlling for other cognitive and linguistic variables and the autoregression. The results suggested a reciprocal developmental relationship between children's compounding awareness and vocabulary knowledge for Chinese children, a finding that informs current models of the relationship between morphological awareness and vocabulary knowledge.

## Introduction

Vocabulary knowledge has been found to be an important indicator of language development and to play an important role in reading comprehension and children's school achievement (Ouellette, 2006; Dickinson and Porche, 2011; Zhang et al., 2013). Children's morphological awareness, which refers to “the ability to reflect upon and manipulate morphemes and employ word formation rules in one's language” (Kuo and Anderson, 2006, p. 161), may be the underlying skill that is particularly associated with vocabulary growth (McBride-Chang et al., 2005, 2008; Chen et al., 2009; Kieffer and Lesaux, 2012; Liu et al., 2013; Sparks and Deacon, 2015). Due to the characteristics of Chinese, Packard (2000) proposed that compounding morphological awareness is salient for Chinese children's language and literacy development. Several studies have shown that morphological awareness is correlated with vocabulary knowledge beyond control variables across languages (e.g., Ku and Anderson, 2003; McBride-Chang et al., 2006; Nagy et al., 2006; Liu and McBride-Chang, 2010). With increased exposure to complex print and spoken words, children's vocabulary knowledge growth facilitates the ability to identify and operate the morphemes, and also the development of morphological awareness contributes to their vocabulary learning. Thus, we speculated that the developmental relationship between morphological awareness and vocabulary knowledge would be reciprocal. Consequently, using a multiwave longitudinal design, the goal of the present study was to explore whether compounding awareness supports development in vocabulary knowledge and vice versa among Chinese children (e.g., McBride-Chang et al., 2008). Understanding the direction of developmental relationship between Chinese children's compounding awareness and vocabulary knowledge has both theoretical and applied value for instruction.

An increasing body of research provides evidence for the close relationship between morphological awareness and vocabulary knowledge for both English speakers (McBride-Chang et al., 2005; Nagy et al., 2006; Ramirez et al., 2014) and Chinese speakers (Ku and Anderson, 2003; McBride-Chang et al., 2006; Chen et al., 2009; Liu and McBride-Chang, 2010; Liu et al., 2013). Ku and Anderson (2003) found the strong relationships of morphological awareness to vocabulary knowledge in Chinese-speaking and English-speaking students. In addition, Chen et al. (2009) examined the associations of compounding awareness to vocabulary acquisition among first and second graders in Mainland China. Results indicated that compounding awareness explained unique variance in vocabulary with age, phonological awareness and RAN controlled. Similarly, Liu and McBride-Chang (2010) found that compounding awareness significantly explained unique variance in vocabulary knowledge. A recent study (Liu et al., 2013) examined the associations of compounding awareness to vocabulary knowledge among Hong Kong Chinese children. The results showed that compounding awareness was uniquely associated with vocabulary. Taken together, these results demonstrate that morphological awareness is closely related to vocabulary knowledge.

To our knowledge, there was three studies directly investigated the temporal direction of the relationship between morphological awareness and vocabulary knowledge (McBride-Chang et al., 2008; Kieffer and Lesaux, 2012; Sparks and Deacon, 2015). However, the results were mixed. One study found the reciprocal relationship, while the other two studies found no reciprocal relationship. In a study of Mandarin groups in which compounding morphological awareness is prevalent, McBride-Chang et al. (2008) examined the relationship between morphological awareness and vocabulary knowledge. The results showed that morphological awareness predicted unique variance in vocabulary knowledge a year later for Chinese 4-year olds, beyond the autoregression and the effects of reading-related skills. Moreover, the reverse relationship was found, that is, vocabulary knowledge also predicted subsequent morphological awareness with the autoregression controlled. Two other studies uncovered evidence for no reciprocal relationship between morphological awareness and vocabulary knowledge. Kieffer and Lesaux (2012) assessed Spanish-speaking language minority learners from fourth through seventh grade annually on English derivational morphological awareness and vocabulary knowledge. Using a parallel latent growth modeling, results found that initial (fourth-grade) level of morphological awareness was not correlated with later growth rate (from fourth-grade to seventh-grade) in vocabulary knowledge, and initial (fourth-grade) level of vocabulary knowledge was not correlated with later growth rate (from fourth-grade to seventh-grade) in morphological awareness. Sparks and Deacon (2015) assessed monolingual English-speaking children longitudinally from Grades 2 to 3. Using cross-lagged panel modeling with autoregression controlled, results found that children's morphological awareness at grade 2 predicted vocabulary knowledge at grade 3, but vocabulary knowledge assessed at grade 2 did not predict morphological awareness at grade 3.

The inconsistencies among the results of these three studies emphasize the need for further research and motivate the current study. In addition, it is unclear whether the reciprocal relationship found in McBride-Chang et al. (2008) extends to elementary school students. The goal of the present study, therefore, was to explore the direction of the developmental relationship between compounding awareness and vocabulary knowledge among Chinese elementary school children.

Several control variables, such as non-verbal reasoning, working memory, phonological awareness, orthographic awareness, and rapid automatized naming, were included in our study to minimize the possibility that any uncovered relationships are the results of extraneous variables. The general reasoning ability, such as non-verbal reasoning, certain cognitive skills, such as working memory and phonological awareness, and some other reading-related cognitive factors, such as orthographic awareness and rapid automatized naming, are associated with children's early vocabulary growth and morphological awareness (Ho et al., 2004; Gathercole, 2006; Stokes and Klee, 2009; de Jong, 2011; Pan et al., 2011; Li et al., 2012). Thus, with these variables controlled, we investigated the developmental relationship between compounding awareness and vocabulary knowledge.

The aim of the present study was to investigate the developmental relationship between compounding awareness and vocabulary knowledge from grades 1 to 2 in Chinese children using a multiwave longitudinal design. Univariate latent growth modeling was used to separately estimate children's initial states and growth rates in compounding awareness and vocabulary knowledge (Curran and Hussong, 2003). After growth was modeled separately for compounding awareness and vocabulary knowledge, a bivariate model was combined to examine the direction of the developmental relationships between compounding awareness and vocabulary knowledge from grades 1 to 2 (Curran and Hussong, 2003). The bivariate model controlled for non-verbal IQ, working memory, phonological awareness, orthographical awareness, and rapid automatized naming on the initial levels and growth rates of the two skills. The model also controlled for the autoregressors which were the paths from the initial levels to growth rates of the two skills. On the basis of the available literature, we sought to test the hypothesized model in which (a) the initial level and growth rate in compounding awareness were allowed to be correlated with the level and growth rate in vocabulary knowledge, (b) the initial level of compounding awareness was allowed to predict the growth rate of vocabulary knowledge from grades 1 to 2, and (c) the initial level of vocabulary knowledge was allowed to predict the growth rate of compounding awareness from grades 1 to 2.

## Materials and methods

### Participants

This study was conducted over the period from October 2012 to April 2014. The study was approved by the local ethical committee of Beijing Normal University. Written informed consent was obtained from school principals, classroom teachers and parents of all of these children for their child's participation prior to data collection.

A total of 149 first grade children (69 girls; mean age at the first testing was 74.92 months) were recruited from two urban elementary schools in Shanxi, China and followed up from grades 1 to 2. All of the children were native Chinese speakers and did not have any severe reading or linguistic developmental delays according to teacher's report.

The children were tested four times between grades 1 and 2: fall (Time 1; *n* = 149) and spring (Time 2; *n* = 146) of the first grade, and fall (Time 3; *n* = 127) and spring (Time 4; *n* = 128) of the second grade. Attrition analyses indicated no significant difference in compounding awareness, vocabulary knowledge, or any of the control variables measured at T1. Missing data were handled using maximum likelihood estimation with robust standard errors in Mplus 7.11 (Muthén and Muthén, 1998-2012) during model estimation.

### Measures and procedure

A total of seven tests were administered in the present study. Nonverbal IQ, working memory, phonological awareness, orthographical awareness, and rapid automatized naming were tested at T1. Compounding awareness and vocabulary knowledge were tested at each time point from T1 to T4. Compounding awareness, vocabulary knowledge, working memory, phonological awareness, and rapid automatized naming were tested individually. Non-veral IQ and orthographical awareness were tested in groups. All the tests were administered by trained testers.

### Compounding awareness

Children's compounding awareness was assessed with the compound production task modeled after the previous research (Liu and McBride-Chang, 2010; Liu et al., 2013), which required children to produce a novel word to correspond to the meaning conveyed by an aurally presented question/scenario. For example, “What should we call a bird like a frog?” The best answer was 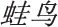 (*wa1 niao3, frog-bird*). This task contained 8 practice items and 20 test items, which were listed in the order of increasing difficulty. Two elementary teachers were required to judge the words in these items to be orally familiar to primary school children. A rating criteria was used to assign scores of 0–3 depending upon the quality of children's responses by two trained psychology students. The rating criteria was based on two aspects: the critical morpheme and the morphological structure. The score 3 was given for the response that included all critical morphemes and a correct and concise structure; the score 2 was allotted for a response that included all critical morphemes and a correct but partially redundant structure, (e.g., 
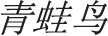
 where 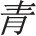 is redundant, or 
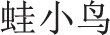
 where 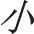 is redundant, for the above example); the score 1 for a response that included all critical morphemes and a correct but completely redundant structure, (e.g., 
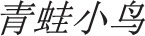
 where 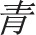 and 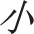 is redundant, for the above example); and the score 0 for a response with missing critical morphemes, or totally unrelated responses or no response. Testing stopped when children failed on five consecutive items. The inter-rater reliability for each time point was 0.95, 0.97, 0.96, and 0.96, respectively. Discrepancies were resolved in discussion.

### Vocabulary knowledge

We assessed children's vocabulary knowledge with a vocabulary definitions task as in the previous research (McBride-Chang et al., 2008), which required children to explain or define the words that were orally presented by the tester. This task has been suggested to be appropriate to participants in the present study as in the previous research (Song et al., 2015). The task contained 1 practice item and 32 test items, which were listed in the order of ascending conceptual difficulty and decreasing word frequency. Children's answers were rated on a scale of 0–2 by two trained experimenters. The score 2 was allotted for the response that fairly completely explained the meaning of the word; the score 1 for an incomplete answer that partially described the meaning of the word; and the score 0 for a totally irrelevant explanation of the word. Testing stopped if a child failed on five consecutive items. The inter-rater reliability for each time point was 0.91, 0.90, 0.93, and 0.93, respectively. Discrepancies were resolved in discussion.

### Non-verbal IQ

Children's non-verbal IQ was assessed with Raven's Standard Progressive Matrices (Zhang and Wang, 1985). The test contained 60 items. The maximum score on the Raven's Matrices was 60.

### Working memory

We assessed children's working memory with digit short-term memory task, which required repetition of digits in backward condition. Longer strings of digits were orally presented to the children at the speed of one digit per second as the task progressed. The length of digit strings ranged from 3 to 12. There were two items for each length. The total score was 10; 0.5 point was given for each digit string correctly recalled.

### Phonological awareness

Phoneme deletion task was used to test children's phonological awareness. Children were required to produce a new syllable by deleting one phoneme from a monosyllabic Chinese word (e.g., /cha1/, with the /ch/ taken away would be /a1/). There were 4 practice items and 12 test items. A correct answer was given 1 point.

### Orthographical awareness

This task asked children to judge and indicate for each with “√” or “×,” whether each of the 90 printed characters could be a real Chinese character. Of the 90 items, 15 items were scrambled strokes, 15 items were position errors, 15 items were radical errors, and 45 items were pseudo characters as fillers. A correct answer was given 1 point, and the total scores were 45 without the fillers.

### Rapid automatized naming (RAN)

It was measured using the rapid digit naming test as in the previous research (Shu et al., 2006). It consisted of 5 different digits repeated randomly five times. Children were required to name the digits as accurately and rapidly as possible twice. Mean time in seconds was calculated.

## Results

### Preliminary analyses

Table 1 presented means, standard deviations, the internal consistency and correlations of all measures used in this study. All the measures had relatively high reliabilities (ranged from 0.74 to 0.92). Obvious increases with time were observed in compounding awareness and vocabulary knowledge. Specifically, vocabulary knowledge and compounding awareness were correlated significantly at each time point, ranging from 0.42 to 0.54. Among the control variables, non-verbal IQ was significantly positively correlated with vocabulary knowledge and compounding awareness at each time point. Working memory was significantly positively correlated with compounding awareness at T1. Phonological awareness was significantly positively correlated with vocabulary knowledge at each time point and compounding awareness at T3 and T4. Orthographical awareness was significantly positively correlated with vocabulary knowledge at each time point and compounding awareness at each time point except T1. RAN was significantly correlated with vocabulary knowledge at T2, T3, and T4.

**Table 1:** **Means, standard deviations, and reliability of and correlations among all variables**.

**Variables**	***M***	***SD***	**Reliability (alpha)**	**1**	**2**	**3**	**4**	**5**	**6**	**7**	**8**	**9**	**10**	**11**	**12**
1.T1_Vocabulary knowledge	8.62	5.13	0.74	1											
2.T2_Vocabulary knowledge	10.69	5.74	0.78	0.71^**^	1										
3.T3_Vocabulary knowledge	13.51	6.36	0.87	0.64^**^	0.67^**^	1									
4.T4_Vocabulary knowledge	14.81	6.39	0.76	0.55^**^	0.50^**^	0.62^**^	1								
5.T1_Compounding awareness	9.57	8.97	0.83	0.42^**^	0.40^**^	0.47^**^	0.33^**^	1							
6.T2_Compounding awareness	15.36	11.64	0.85	0.47^**^	0.50^**^	0.45^**^	0.44^**^	0.64^**^	1						
7.T3_Compounding awareness	20.94	10.81	0.80	0.47^**^	0.52^**^	0.54^**^	0.49^**^	0.60^**^	0.65^**^	1					
8.T4_Compounding awareness	25.87	10.52	0.78	0.46^**^	0.49^**^	0.48^**^	0.54^**^	0.47^**^	0.62^**^	0.70^**^	1				
9.T1_Nonverbal IQ	28.06	9.31	0.91	0.40^**^	0.37^**^	0.35^**^	0.31^**^	0.28^**^	0.30^**^	0.28^**^	0.28^**^	1			
10.T1_Working memory	1.26	0.82	0.92	0.13	0.09	0.02	0.12	0.19^*^	0.07	0.02	0.16	0.24^**^	1		
11.T1_Phonological awareness	6.05	3.83	0.88	0.29^**^	0.25^**^	0.20^*^	0.32^**^	0.14	0.09	0.26^**^	0.30^**^	0.18^*^	0.34^**^	1	
12.T1_Orthographical awareness	25.80	8.74	0.92	0.25^**^	0.20^*^	0.17^*^	0.18^*^	0.12	0.16^*^	0.22^*^	0.19^*^	0.29^**^	0.14	0.11	1
13.T1_Rapid automatized naming^a^	15.71	5.17	0.84^b^	−0.10	−0.22^*^	−0.18^*^	−0.22^*^	−0.15	−0.07	−0.09	−0.06	−0.03	−0.11	−0.18^*^	−0.06

*Ns = 127 - 149*.

a *In seconds*.

b *test-retest reliability*.

* *p < 0.05*,

** *p < 0.01, two-tailed*.

### Univariate latent growth modeling

Latent growth modeling was performed using Mplus 7.11 (Muthén and Muthén, 1998-2012). To evaluate the model fit, we reported chi-square values, the chi-square values to *df* ratio, comparative fit index (CFI), Tucker-Lewis index (TLI), root mean square error of approximation (RMSEA), and standardized root mean square residual (SRMR). According to recommendations by Kline (2011), a good model should have a χ^2^ to *df* ratio smaller than 2, CFI and TLI values larger than 0.95. RMSEA and SRMR values are smaller than 0.05 reflecting good fit and smaller than 0.08 suggesting satisfactory fit.

Univariate latent growth modeling was used to separately ascertain children's initial level (i.e., intercept) and growth rate (i.e., slope) parameters of vocabulary knowledge and compounding awareness. As is customary in latent growth modeling, the intercept loadings of the observed variables across T1–T4 were fixed to 1; the four slope loadings were fixed to 0, 1, 2, and 3 for the first, second, third, and fourth measurement occasion, respectively. The model (Model 1) of vocabulary knowledge showed poor fit, χ^2^(*df* = 5) = 11.75, *p* = 0.04 (χ^2^/*df* = 2.35), CFI = 0.97, TLI = 0.97, RMSEA = 0.10 (90% CI = 0.02 − 0.17), SRMR = 0.04. Examination of the modification indices suggested that the estimated residual terms between the measurements at T2 and T3 were allowed to correlate. The results showed that the model (Model 2) had an acceptable fit, χ^2^(*df* = 4) = 7.10, *p* = 0.13 (χ^2^/*df* = 1.78), CFI = 0.99, TLI = 0.98, RMSEA = 0.07 (90% CI = 0.00 − 0.16), SRMR = 0.04. Significant Chi square difference was found when comparing Model 1 to Model 2, χ^2^_diff_ (*df* = 1, *N* = 149) = 4.65, *p* = 0.03. The results indicated that the modification model (Model 2) is better than Model 1. Thus, we chose this model (Model 2) as our final model. For compounding awareness, the model showed satisfactory fit, χ^2^(*df* = 5) = 8.33, *p* = 0.14 (χ^2^/*df* = 1.67), CFI = 0.99, TLI = 0.98, RMSEA = 0.07 (90% CI = 0.00 − 0.14), and SRMR = 0.05.

Table 2 presented parameter estimates for the latent growth modeling of the two variables. The parameter estimates of the models for vocabulary knowledge and compounding awareness indicated a relatively low initial level at T1, followed by positive and significant growth rate from T1 to T4. There were significant individual differences in the initial levels and growth rates, indicating that children differed in their initial level and growth rate in vocabulary knowledge and compounding awareness. The estimated correlation between the initial level and slope of vocabulary knowledge and compounding awareness was significant, respectively, suggesting that students with a lower initial level of compounding awareness and vocabulary knowledge had faster growth rates.

**Table 2:** **Parameter estimates for univariate latent growth models of vocabulary knowledge and compouding awareness**.

**Variable**	**Initial level**		**Growth rate**		**Covariance between initial level and growth rate**	
	**Mean Coeff. (*SE*)**	**Variance Coeff. (*SE*)**	**Mean Coeff. (*SE*)**	**Variance Coeff. (*SE*)**	**Coeff. (*SE*)**	**Stand**.
Vocabulary knowledge	8.62^***^(0.42)	23.82^***^(3.75)	2.18^***^(0.16)	1.65^**^(0.60)	−2.01(1.20)	−0.32^*^
Compounding awareness	9.65^***^(0.74)	68.73^***^(10.54)	5.59^***^(0.29)	7.51^**^(2.05)	−6.50(3.79)	−0.29^*^

*Coeff., coefficient; Stand., standardized coefficient*.

* *p < 0.05*,

** *p < 0.01*,

*** *p < 0.001, two-tailed*.

### Bivariate latent growth modeling

**Figure 1:**
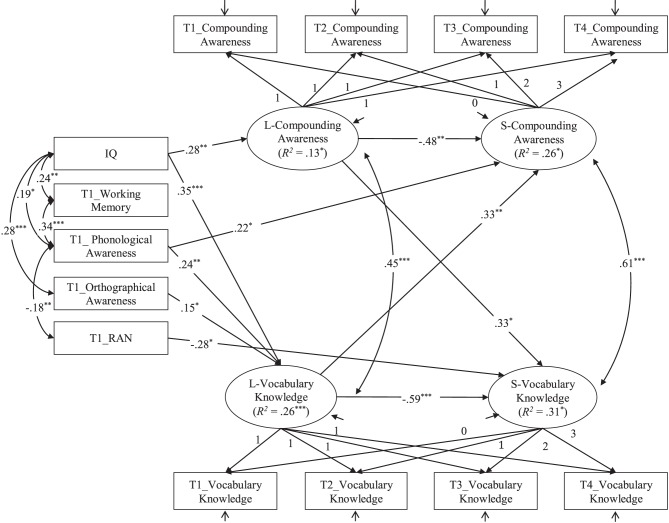
**Bivariate latent growth model with a completely standardized solution to address the direction of developmental relationship between compounding awareness and vocabulary knowledge after controlling for IQ, working memory, phonological awareness, orthographical awareness, rapid automatized naming, and the autoregressions**. *N* = 149. Non-significant paths are not shown here to simplify the representation of the model. ^*^p < 0.05, ^**^p < 0.01, ^***^p < 0.001, two-tailed.

Having successfully modeled the growth of compounding awareness and vocabulary knowledge separately, it was then possible to investigate the developmental relationship by modeling them simultaneously using a bivariate latent growth modeling, while controlling for the other cognitive and linguistic variables and the autoregressors. In the bivariate latent growth modeling, the critical additional parameters, which were indicators of the level of one construct could predict the subsequent growth rate of the other construct, were permitted to estimate. We also calculated covariances between compounding awareness and vocabulary knowledge levels and growth rates in the model. All control variables were permitted to correlate with each other, and all control variable paths to the initial levels and growth rates of the two constructs were calculated. To control the autoregressors, the paths from the initial level to growth rate of compounding awareness and vocabulary knowledge were also calculated. The model showed a good fit, χ^2^(*df* = 41) = 56.56, *p* = 0.05 (χ^2^/*df* = 1.38), CFI = 0.97, TLI = 0.96, RMSEA = 0.05 (90% CI = 0.00 − 0.08), and SRMR = 0.04. Figure 1 showed this model with the estimates of the standardized path coefficients. The initial level of compounding awareness positively predicted the growth rate of vocabulary knowledge (standardized β = 0.33, *p* < 0.05), and the initial level of vocabulary knowledge positively predicted the growth rate of compounding awareness (standardized β = 0.33, *p* < 0.01) after controlling for the other cognitive and linguistic covariates and the autoregressions. The significant and positive crossed paths between initial levels and growth rates indicated that growth in vocabulary knowledge was accounted for in part by level of compounding awareness and vice versa. The positive correlations between initial level of compounding awareness and initial level of vocabulary knowledge (*r* = 0.45), and between growth rate of compounding awareness and growth rate of vocabulary knowledge (*r* = 0.61) indicated the fact that the initial levels and growth rates in compounding awareness and vocabulary knowledge were positively correlated.

## Discussion

Latent growth modeling was conducted to examine the direction of developmental relationship between compounding awareness and vocabulary knowledge. We firstly modeled the development of compounding awareness and vocabulary knowledge separately, which was to ascertain the univariate growth model for further examining the temporal direction of the relationship between them. The present results confirmed the earlier studies that show evidence of positive associations in initial levels and growth rates of the two variables (McBride-Chang et al., 2008; Kieffer and Lesaux, 2012), demonstrating that compounding awareness and vocabulary knowledge were developmentally intertwined. We further added to prior research by testing the temporal direction of compounding awareness and vocabulary knowledge, finding evidence of the predictive associations between initial level of compounding awareness and subsequent development in vocabulary knowledge and vice versa. The results demonstrated that there was a reciprocal developmental relationship between compounding awareness and vocabulary knowledge, even after controlling for other cognitive and linguistic factors and the autoregression. Our results were consistent with those of McBride-Chang et al. (2008), who also found the bootstrapping relationship between compounding awareness and vocabulary knowledge, while were inconsistent with the single direction from morphological awareness to vocabulary knowledge reported by Sparks and Deacon (2015) and the correlation but no direction reported by Kieffer and Lesaux (2012). The reason may be due to the characteristic of the Chinese and English. Both Sparks and Deacon (2015) and Kieffer and Lesaux (2012) examined the relationship between inflectional and derivational morphological awareness and vocabulary knowledge, while McBride-Chang et al. (2008) investigated the relationship between compounding morphological awareness and vocabulary knowledge. More specifically, the current study goes beyond the study by McBride-Chang et al. (2008) by extending the reciprocal relationship to the elementary school years.

Our results revealed that the initial level in compounding awareness at the beginning of grade 1 was a significant predictor of the growth rate of vocabulary knowledge from grades 1 to 2, even after other cognitive and linguistic variables and the autoregressor were statistically controlled. Although previous studies have confirmed that morphological awareness is important for vocabulary knowledge in Chinese children, both concurrently and longitudinally (McBride-Chang et al., 2006, 2008; Chen et al., 2009; Liu and McBride-Chang, 2010; Liu et al., 2013), no previous research, however, has taken growth rate into consideration. Latent growth modeling has allowed us to identify the role of the initial level of compounding awareness in the growth rate of vocabulary knowledge. It seems that children are able to use their compounding awareness and to identify and manipulate the morphemes within compound words to make progress in acquiring vocabulary knowledge and support the development of their vocabulary knowledge. In the present study, compounding awareness was assessed by compounding production task, which required children to extract the key morphemes and combine them into a compound word. Therefore, compounding awareness can provide extra cues to help children learn new compound words and infer the meaning of compound words (Kuo and Anderson, 2006; Liu et al., 2013). In this way, compounding awareness facilitates children's vocabulary knowledge growth.

In the present study, we also found that children's initial vocabulary knowledge significantly predicted the growth of compounding awareness. It seems that children who have more vocabulary size can use their understanding of the meaning of the words better to gain more from their experiences (Kuo and Anderson, 2006; McBride-Chang et al., 2008; Chen et al., 2009; Ramirez et al., 2014). In other words, the vocabulary knowledge experiences provides an opportunity for children to develop abstract understanding of key morphemes and extract the structure of compounding word, and then support the growth of compounding awareness (McBride-Chang et al., 2007). For example, children can develop abstract understanding of the key morpheme 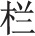 (lan2, fence) from the words 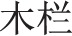 (mu1 lan2, wooden fence), 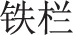 (tie3 lan2, iron fence), and 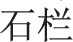 (shi2 lan2, stone fence). In addition, they can extract the compounding structure rules in this example, which is the first morpheme modifies the second one.

As children's literacy and reading development, the relationships between compounding awareness and vocabulary knowledge become cumulatively reciprocal. In other words, children's ability of morphological analysis makes them decompose complex words to infer the meaning of morphologically complex words from the morphemes and compound structure. In turn, learning more words makes them infer the meaning of critical morphemes to avoid confusing many homophones better and the compound structure from their experiences. It seems that compounding awareness and vocabulary knowledge are on the basis of each other and developmentally intertwined.

The work presented here had some limitations that might be addressed in future research. First, even though the present study was longitudinal in nature, our research design could not make causal inferences. Experimental or intervention studies should be necessary to test the temporal direction of developmental relationships between compounding awareness and vocabulary knowledge in future research.

Second, although compounding awareness is prevalent in Chinese, there are a large number of homophones and homographs in Chinese languages. The structure of morphological awareness includes homophone awareness, homograph awareness, and compounding awareness in Chinese (Liu et al., 2013). Future studies could examine the relationship between different components of morphological awareness and vocabulary knowledge systematically among older students.

Third, children received the same versions of compound awareness and vocabulary knowledge tasks across 4 time points in our study. Although we have addressed some steps to ensure the children could not remember the tests. And the 6-month interval is sufficient and appropriate for the present study. The results still may be confounded with memory for children received the same testing materials. Future studies should develop the parallel tests for compounding awareness and vocabulary knowledge tasks in multiwave longitudinal studies to reduce the item sensitivity, tap children's attention and reach the more resonable conclusions.

Despite these limitations, however, the findings have important implications for research and practice. First, unraveling the direction of developmental relationships between compounding awareness and vocabulary knowledge may contribute to theories of Chinese literacy acquisition. Using the multi-wave longitudinal design, our analyses suggest that the developmental relationships between compounding awareness and vocabulary knowledge are reciprocal with most stringent controls of other variables. In addition, our findings may shed light on the instructional practices for early children's language and reading developments. Hence, we argue that to make compounding awareness instruction aimed at improving their vocabulary knowledge performance and vice versa.

In conclusion, our results provide empirical evidence that children's compounding awareness is a predictor of the growth of vocabulary knowledge and children's vocabulary knowledge also predicts the growth of compounding awareness. The findings demonstrate the reciprocal developmental relationship between morphological awareness and vocabulary knowledge for Chinese children.

## Author contributions

Conception and design of the study: YC, XW Acquisition, analysis, and interpretation of data: YC, LL, XW Drafting the work and revising it critically for important intellectual content: YC, LL, XW.

### Conflict of interest statement

The authors declare that the research was conducted in the absence of any commercial or financial relationships that could be construed as a potential conflict of interest.
